# A Description of Female Genital Mutilation and Force-Feeding Practices in Mauritania: Implications for the Protection of Child Rights and Health

**DOI:** 10.1371/journal.pone.0060594

**Published:** 2013-04-09

**Authors:** Nacerdine Ouldzeidoune, Joseph Keating, Jane Bertrand, Janet Rice

**Affiliations:** 1 Tulane University, Payson Center for International Development, New Orleans, Louisiana, United States of America; 2 Tulane University School of Public Health & Tropical Medicine, Department of Global Health Systems & Development, New Orleans, Louisiana, United States of America; 3 Tulane University School of Public Health and Tropical Medicine, Department of Biostatistics and Bioinfomatics, New Orleans, Louisiana, United States of America; Indiana University and Moi University, United States of America

## Abstract

**Objectives:**

To establish the prevalence of female genital mutilation (FGM) and force feeding (*gavage*) practices among children in Mauritania; to investigate factors related to FGM and *gavage* practices and attitude in Mauritania; and to explore implications related to the protection of children’s rights and welfare.

**Methods:**

Data from the Mauritania 2000–2001 DHS were used in this analysis. Data were collected from men and women about their attitude toward the continuation of FGM and *gavage;* women only were asked if they ever experienced one of these practices. Chi-square statistics were used to investigate differences in attitude and practice of FGM and *gavage* by demographic characteristics. Binary logistic regression was used to identify socio-demographic factors related to FGM and *gavage* outcomes.

**Findings:**

The overall prevalence of FGM was 77% but varied depending on ethnicity. The majority of both female and male respondents favored the continuation of the practice (64% and 70%, respectively). Almost a quarter (23%) of women reported being force fed as a child and 32% of women and 29% of men approved the continuation of the practice. *Gavage* is almost exclusively practiced among Arabs.

**Conclusion:**

The practice of both FGM and *gavage* is ongoing, although the prevalence and attitude towards both appears to vary as a function of ethnicity, wealth, education, marital status, and age. Contextually relevant intervention and enforcement strategies are needed to challenge these cultural norms and protect the rights and welfare of children in Mauritania.

## Introduction

According to Article 5 of the Universal Declaration of Human Rights, “no one shall be subjected to torture or to cruel, inhuman or degrading treatment or punishment.” [1]. This paper presents evidence of continued female genital mutilation (FGM) and *gavage* (force-feeding of adolescent females) in Mauritania. While these practices are culturally sanctioned in the Mauritanian context, the harming of genital organs of any child (including harm resulting from the practice of *gavage* and FGM) is illegal under the child protection penal code; penalties range from 1–3 years imprisonment and heavy fines. However, the law does not specify FGM or *gavage* as explicit illegal practices, although both practices have the potential to result in pain and psychological suffering on the girl. Further, FGM has been linked to the development of feelings of incompleteness, loss of self-esteem, depression, chronic anxiety, phobias, panic and psychotic disorders [2]. No information was found on the psychological effects of gavage; this void in the literature represents an important area for further investigation in Mauritania.

Whereas FGM has been documented in numerous sub-Saharan Africa (SSA) countries [3]–[6] and is widely recognized by the international community as a human rights violation and public health risk to women in countries where it is practiced [2], *gavage* is somewhat unique to a few African countries and relatively unknown to many, including experienced international public health professionals. Given that *gavage* has been linked to child marriage and violence against young girls [7]–[9], and both FGM and gavage are a public health concerns and violate the rights and welfare of children, more needs to be done to understand the practices before effective challenges to these cultural norms are launched.

### Female Genital Mutilation

FGM, also known as female genital cutting describes all procedures that involve complete or partial removal of the external female genitalia, or injury to the female genital organs for non-therapeutic reason. It has no known health benefits and may harm girls and women permanently [3], [10]–[15]. There are four general types of FGM practiced [14], although the type and extent varies by country and culture: 1) Clitoridectomy, where the clitoris is either partially or totally removed; 2) Excision, where the clitoris and labia are either partially or totally removed; 3) Infibulation, which refers to a condition where the vaginal orifice is narrowed by a seal created by cutting and transposing the labia majora or the labia minora; and 4) other practices that include cauterizing, incising, pricking, scraping or piercing the genital areas.

FGM is generally believed to curb a woman’s sexual desire and many assume it is required for religious reasons; it is also done to confer social acceptance on the family, as well as a rite of passage from childhood to adulthood [16]–[20]. The majority of FGM practiced in Mauritania are types I and II; FGM is typically done at a very early age ranging from a few weeks old to 3 years of age.

### Gavage

*Gavage* is a French term that literally means “force feeding”; force-feeding is a method used to fatten geese for *foie gras*. In Mauritania, *gavage* is known as “Leblouh”. The practice of fattening girls has potentially harmful consequences related to reduced movement and increased risk of developing cardiovascular diseases [8]. According to the World Health Organization (WHO), approximately three million adult deaths occur every year resulting from obesity. As well, a significant proportion of diabetes, heart diseases and cancers are attributed to obesity or being overweight. [21].

*Gavage* is predominantly practiced among Arab families in Mauritania; while there are also examples of pre-marriage “fattening houses” for women among other ethnic groups in West Africa, this fattening is typically done at a later age, is done only for the purpose of the immediate marriage, and is usually consensual. Mauritania ranks number one on a list of countries that celebrate female obesity; this celebration poses human rights violations if girls are being forced against their will to fatten, in addition to the public health concerns related to obesity. A second problem relates to a lack of financing to combat the negative physical and psychological health effects of obesity in Mauritania [22]–[23].

As the social norms for marriage favor young girls that have a large amount of body fat, families often force young girls (sometimes as early as 6 years old) to consume large quantities of food and milk to increase their weight, and thus their chances for marriage at an early age. The use of pills (e.g. steroids) to gain weight has also been reported in Mauritania. Thin women are believed to be unhealthy, whereas overweight women are perceived as far more desirable [24]–[25]. Further, the large size of these force-fed girls creates an illusion that they are physically mature and ready for marriage; this creates the potential for reproductive health and psychological problems later in life if the girl has not yet reached maturity.

There is a little reference as to when force-feeding of girls began in Mauritania. Some historians believe that the practice dates back centuries when most of the white Mauritanians, Arabs, and Berbers were nomads; in nomadic society the obesity of women was seen as a sign of beauty and prosperity and the wives of rich men would often not work, preferring instead to sit in tents while black slaves tended to household chores. [26].

Ostensibly, FGM and *gavage* have similarities. Both are practiced on girls, and mothers or grandmothers often perpetuate these practices. Both also have strong gender implications relating to harm performed in the name of increasing a girl’s desirability for marriage or social acceptance. And, both represent serious human rights violations, as these practices produce physical suffering and have implications for long-term psychological well-being.

There is a dearth of published literature or statistics on public health problems in Mauritania in general, and even less on these two practices specifically. This paper constitutes the first published report to examine these two practices in terms of prevalence, prevailing attitudes and acceptance, and factors associated with their use in Mauritania. While the data used are almost a decade old, the 2001 demographic and health (DHS) survey constitutes the only known population-based survey to collect data on both FGM and *gavage*. The existence of two such harmful practices to girls in a single country highlights the importance of surveillance and investigations into these practices simultaneously. As such, the objectives of this paper are 1) to document the prevalence of FGM and *gavage* in Mauritania, 2) to investigate gender differences in attitude towards these practices, and 3) identify factors associated with FGM and *gavage* practices. The results presented herein are intended to increase awareness and serve as a platform for developing culturally relevant prevention mechanisms in Mauritania and elsewhere with the long-term goal of improving the reproductive and psychological health of girls.

## Methods

### Study Site

Mauritania is a country located in Northwest Africa between Morocco, Western Sahara and Senegal. It is one of the poorest and least developed countries in the world. In 2011, the country’s Human Development Index (HDI) was very low (0.453), ranking 159^th^ out of 182 countries [27]. Over half (58.6%) of the population lives in rural areas; the dispersed settlement pattern often poses health care access challenges for many communities. Of the country’s four ethnic groups (Arab, Poular, Soninke, and Wolof), Arabs are by far the largest. [8]. Life expectancy is 56 years for males and 60 years for females. The maternal mortality rate is 550 deaths per 100,000 live births. Only 4% of the central government expenditure is allocated to health, representing 2.2% of gross domestic product [23]. Mauritania ranks 114^th^ out of 135 countries on the Gender Gap index [28].

### Study Design and Sample Size

Data were obtained from the Mauritania 2001 Demographic and Health survey (DHS) [8]. This was a secondary analysis of DHS data; the Mauritanian government gave permission to use these data. The Tulane University Institutional Review Board (IRB) reviewed and approved the analysis only, as Tulane University researchers were not involved with data collection or study design. The DHS survey was approved by the Mauritanian Government (National Direction of Statistics).

The DHS targets women of reproductive age and therefore a statement about children is not needed. Due to high illiteracy rates in the area, verbal consent and assent, in lieu of written consent and assent, were obtained during the collection of data. No data were collected from respondents who did not give permission to participate. All interviews were conducted in private to reduce social desirability bias. All data were collected using a standardized structured questionnaire [8].

The survey used a two-stage cluster design to produce a probability sample, representative of the entire country; data were collected on women of reproductive age (15 to 49 years old), and men (15 to 59 years old). A total of 6,149 households were successfully surveyed; only 1.3% of households approached refused to participate. Women between the ages of 15 and 49 years old in selected households were interviewed individually; a total of 7,728 were interviewed, with 3.5% of eligible women refusing to participate. Forty-percent (40%) of households were selected for male interviews; a total of 2,191 men between the ages of 15 and 59 years old were interviewed, with 11.9% of eligible men refusing to participate.

### Data Analysis

STATA 9.0 (STATA Corporation, College Station, TX) was used for all data analyses. Descriptive statistics were used to summarize 2001 DHS household survey data by demographic characteristics. Chi-square test statistics were used to explore differences between demographic characteristics and the following 6 outcomes: attitude about continuing the practice of FGM (among men and women separately), attitude towards continuing the practice of *gavage* (among women and men separately), the prevalence of FGM among women, and the prevalence of *gavage* among women.

Individual level demographic characteristics included age and education. Household level demographic characteristics included wealth, as measured by a household asset index created using a principal components analysis of household assets (e.g. electricity, radio, television, refrigerator, vehicle and animals) [29]; place of residence (urban versus rural and region); and ethnicity (Note: in some analyses, ethnicity categories were either dropped or collapsed to increase the sample size within categories). Lifestyle binary variables included marital status and working status. While we recognize that other variables may also be important, the socioeconomic and demographic variables described above were chosen as a starting point for investigations.

Logistic regression models were used to test whether socio-demographic factors were associated with the 6 outcomes related to attitudes and practices of FGM and *gavage*. Regressions using FGM outcomes excluded region of residence due to collinearlity with residence in rural versus urban areas. An interaction term between place of residence and working status was included in both of the multivariate models; no other interaction terms tested significant. Regressions on attitude were done among respondents who had heard of FGM or *gavage*. A second set of logistic regression models were used to test for factors associated with the prevalence of both FGM and *gavage* among women who have heard of the respective practices. All regressions included only those respondents with complete data and reported knowing of the respective practices. No differences were detected in prevalence or attitudes between those with complete data records and those within incomplete data records.

To control for intra-class correlation at the community level, empirically estimated standard errors were used within logistic regression models, using the village as the cluster unit. Probability weights were used to account for the two-stage cluster sample design used. The probability of committing a type-1 error (alpha) was set at 0.05. Wald statistics and log-likelihood ratios were used to identify variable significance and model fit.

## Results

Table 1 presents data on the socio-demographic profile of the sample. As expected, approximately 75% and 20% of the sample were Arab and Poular, respectively. About a quarter of women were single at the time of the survey, compared to almost half of men. Roughly half of the respondents were from urban areas, with almost 40% living in the capital city of Nouakchott.

**Table 1:** Socio-demographic characteristics of the sample.

	Women (%)	Men (%)
	n = 7,728	n = 2,191
**Ethnicity**		
Arab	76.0	74.5
Poular	16.6	17.7
Soninke	4.0	4.0
Wolof	2.2	2.8
Others	1.0	0.8
**Marital status**		
Single never married	28.6	48.8
Married	58.8	48.9
Divorced and widowed	12.6	2.3
**Place of residence**		
Urban	46.0	56.2
Rural	54.0	43.9
**Wealth**		
Lowest	35.1	28.1
Second	31.2	28.8
Middle	4.0	7.9
Fourth	16.4	20.9
Highest	13.3	14.3
**Education**		
No	30.5	21.1
Koranic	27	19.2
Prim. Education	27.8	26.1
Second. Education	13.6	28.3
High (University)	1.1	5.3
**Age**		
15–19	22.0	22.5
20–24	19.0	14.6
25–29	16.9	13.6
30–34	15.4	11.8
35–39	10.8	10.4
40–44	10.1	11.4
45–49	5.9	6.4
50–54	–	6.1
55–59	–	3.2
**Working Status**		
Not working	70.6	33.5
Working	29.4	66.2

### Knowledge, Attitude, and Prevalence of FGM

In Mauritania, over 70% of women report experiencing FGM [8]. While the extent of this practice varies somewhat as a function of cultural practices and location in Mauritania, it has been reported as widespread [8], [15]. Table 2 presents data on attitudes toward FGM. The vast majority of women and men (92% and 82%, respectively) had heard of FGM. When asked what they perceived to be the advantage of FGM, the most common responses were higher social recognition, fulfilling a religious requirement, and reducing female sexual desire. Over half of both male and female respondents reported that there are no disadvantages to the practice of FGM (53% of women and 58% of men). The majority of both female and male respondents favored the continuation of the practice (64% and 70%, respectively). Interestingly, there was discordance between male and female beliefs that that opposite sex desired the continuation of the practice, with 37% of women reporting that they thought that males wanted to continue the practice and 55% of men reporting that women wanted to continue the practice of FGM. The practice of FGM among respondents in this sample was widespread, with 77% of women reporting that they experienced FGM. Among respondents (both male and female) who had at least one daughter, 71% reported practicing FGM on their daughter.

**Table 2:** Knowledge and attitude toward FGM.

	Women (%)	Men (%)
	n = 7,728	n = 2,191
**Knowledge: Has heard of FGM**		
Yes	91.7	82.2
**Among those that know of FGM**	**n = 7,158**	**n = 1,818**
**What are the advantages of practicing FGM?**		
Social recognition	34.8	29.6
Curbs sexual desire	31.2	25.1
Religious requirement	29.2	41.2
Better for hygiene	18.8	13.0
Better chance to get married	3.8	9.8
Sexual desire of the other sex	1.8	1.8
Other	8.5	10.6
None	21	19.9
**What are the advantages of not practicing FGM?**		
More sexual desire for women	19.7	12.9
Less health problems	10.6	9.7
Avoid suffering	6.9	6.1
More sexual desire for men	5.7	4.9
Less delivery problems	4.4	1.6
Accordance with religion	2.4	3.4
Other	11.9	12.9
None	52.9	58.7
**Belief that FGM curbs sexual desire**		
Yes	38	20.5
No	21.6	14.2
Don’t know	39.1	63.4
**Is the practice of FGM required by your religion?**		
Yes	41.6	35.8
No	29.2	27.5
Don’t know	28.0	34.0
**Should FGM be retained (attitude)?**		
Yes	64.4	70.9
No	22.3	17.7
It depends	7.2	4.5
Don’t know	5.8	6.3
**Do you think (the other sex) wants it to be retained?**		
Yes	37.0	56.1
No	14.6	13.3
It depends	23.2	12.7

* For some questions the columns do not equal 100% due to missing data.

Bivariate results indicated significant differences in attitude (i.e., approval of the continuation of FGM) for both male and female respondents. The most significant differences were observed among women and among men who live in different regions [female (*χ^2^* = 1,260.6, p<0.001), male (245.9, p<0.001)], with both women and men who live in Nouakchott less likely to approve of the continuation of FGM. Women and men in the highest wealth quintile were also less likely to approve of continued FGM [female (*χ^2^* = 550.1, p<0.001)], male (*χ^2^* 103.2, p<0.001)]. Women and men in rural areas were more likely to approve of continued FGM [female (*χ^2^* = 310.5, p<0.001), male (*χ^2^* = 126.9, p<0.001)].

### Knowledge, Attitude, Intention to Practice, and Prevalence of Gavage

In Mauritania, over 20% of women report experiencing *gavage* [7]. Table 3 presents data on attitudes toward *gavage*. Almost all respondents had heard of *gavage* (92%). Approximately a third of respondents reported that this practice increased a girl’s beauty (40% of women and 30% of men) and about a quarter reported that the practice increased the family’s social standing in the community (27% of women and 21% of men). In contrast, nearly 40% of women and 55% of men reported that *gavage* has no advantages. The most common reason given to not practicing *gavage* was improved health. In contrast, approximately a quarter of both men (23%) and women (25%) cited no disadvantages with the practice of *gavage*. Among respondents with at least one daughter, 17% of women and 11% of men reported the intention to practice *gavage*. Almost a quarter (23%) of women reported being force fed as a child; among those with a daughter, 6% of women and 11% of men had already force fed at least one daughter. Over 61% of those who had experienced gavage reported being beaten during the process and almost one-third (29%) reported having their fingers broken to encourage participation. Conversely, among those women who regretted being force-fed, over half (58%) reported health risks as the most common reason; over one-third (34%) reported that being big made it too difficult to work and move. Pain was also a common reason (31%) cited for regret.

**Table 3:** Knowledge, attitudes, and intentions toward the practice of *gavage*.

	Women (%)	Men (%)
	n = 7,615	n = 2,158
**Knowledge: Has heard of ** ***gavage***		
Yes	93.7	91.5
**Among those that have heard of ** ***gavage***	**n = 7,212**	**n = 2,000**
**Advantages of force feeding**		
More beautiful	40.2	30.8
Show the social level	27.1	20.8
More chances for marriage	13.8	3.7
Other	4.4	6.4
None	39.8	54.5
Don’t know	5.8	4.7
**Advantages of not force feeding**		
Better for health	44.8	55.0
Make it easier to work and move	34.7	50.1
More attractive	10.4	9.3
Avoid pain	9.8	11.4
Avoid ugly look when you lose weight	7.0	7.4
Facilitate pregnancy and delivery	3.7	5.0
Avoid stretch marks	3.6	4.7
More chance to get married	2.6	1.3
Sexual pleasure of women	1.5	0.6
Accordance (in line) with religion	0.4	1.4
Other	3.1	2.6
None	24.6	23.7
Don’t know	9.0	7.1
**Among those that do not intend to force feed daughter(s)**	**n = 2,440**	**n = 537**
**Reasons not to force feed:**		
Bad for health	52.8	58.2
Hamper ability to work and move	28.4	33.3
Very painful	11.6	15.9
Very expensive	10.1	13.0
Difficulty for pregnancy and delivery	8.7	8.4
Against Gavage	6.5	22.5
Ugly if you lose weight	5.8	4.0
Stretch marks	5.7	4.2
Less chances for marriage	1.1	1.8
None	19.6	19.4
Others	5.1	6.6
Don’t know	10.9	4.6

* For some questions the columns do not equal 100% due to missing data.

Bivariate results indicated significant differences in attitude (approval of the continuation of *gavage*) for males and females. The most important differences in attitude toward the continuation of *gavage* were observed among women and among men from different ethnic groups, with Arabs more likely to approve of the continuation of *gavage* [female (*χ^2^* = 358.8, p<0.001) male (*χ^2^* = 49.9, p<0.001)]. Men and women from Nouakchott were also more likely to approve of continued *gavage*: [female (*χ^2^* = 351.2, p<0.001), male (*χ^2^* = 374.0, p<0.001)]. Those from the lowest wealth quintile also supported the continued practice of *gavage*: [female (*χ^2^* = 257.2, p<0.001), male (*χ^2^* = 111.1, p<0.001)].

### Multivariate Analysis of Socio-demographic Factors Related to FGM and Gavage

Results from the 3 logistic regressions with FGM attitude and practice outcomes are presented in Table 4. The strongest predictors for approving (attitude) of continued FGM were ethnicity, wealth and education for both male and female respondents; place of residence was also strongly associated with the outcome for male respondents. Respondents reporting non-Wolof ethnic origin were 7.7 (95% CI: 3.76–15.83) and 5.22 (95% CI: 2.51–10.86) times more likely to approve of the continuation of FGM compared to their Wolof counterparts for women and men, respectively. High wealth was negatively associated with approval of the continued practice for both women [O.R. 0.37 (95% CI 0.28–0.50)] and men [O.R. 0.52 (95% CI 0.28–0.93)]. High education also was negatively associated with the approval of FGM continuation for both women [O.R. 0.19 (95% CI 0.11–0.33)] and men [O.R. 0.29 (95% CI 0.16–0.52)]. Not surprisingly, men reporting residence in rural areas were more likely to approve of FGM continued practice [O.R. 2.82 (95% CI 1.09–7.31)]. Women between the ages of 15 and 19 were also more likely to report approval, although age was not a significant factor for men.

**Table 4:** Multivariate analysis of factors related to practice and attitude toward FGM.

	Favorable attitude for continuing FGM		Experienced FGM
	Women (n = 6,074)¥	Men (n = 1,583)	Women (n = 7,048)§
	*Odds Ratio (95% CI)*	*Odds Ratio (95% CI)*	*Odds Ratio (95% CI)*
**Ethnicity**			
Wolof	Reference	Reference	Reference
Other ethnic groups	7.71 (3.76–15.83) **	5.22 (2.51–10.86)**	7.61 (4.56–12.68) **
**Place of residence**			
Urban	Reference	Reference	Reference
Rural	1.20 (0.82–1.75)	2.82 (1.09–7.31)*	1.45(.95–2.20)
**Wealth**			
Lowest	Reference	Reference	Reference
Second	0.98 (0.79–1.21)	0.81 (0.48–1.39)	1.08 (0.85–1.37)
Middle	1.14 (0.79–1.65)	0.84 (0.38–1.87)	0.67 (0.46–0.97)*
Fourth	0.74 (0.58–0.94)*	0.50 (0.29–0.86)*	0.63 (0.48–0.81)**
Highest	0.37 (0.28–0.50) **	0.52 (0.28–0.93)*	0.45 (0.32–0.63)**
**Education**			
No	Reference	Reference	Reference
Koranic	0.68 (0.52–0.88)**	1.18 (0.65–2.16)	1.36 (1.06–1.73)*
Prim. Education	0.60 (0.47–0.76)**	0.67 (0.39–1.17)	0.93 (0.75–1.14)
Second. Education	0.35 (0.27–0.46)**	0.49 (0.29–0.82)**	0.65 (0.52–0.82)**
High (University)	0.19 (0.11–0.33)**	0.29 (0.16–0.52)**	0.69 (0.39–1.23)
**Age**			
15–19	Reference	Reference	Reference
20–24	0.78 (0.64–0.94)**	0.77 (0.47–1.27)	1.00 (0.84–1.20)
25–29	0.67 (0.54–0.83)**	0.69 (0.41–1.15)	0.88 (0.70–1.10)
30–34	0.65 (0.52–0.82)**	0.64 (0.36–1.14)	1.14 (0.89–1.47)
35–39	0.47 (0.37–0.61)**	1.05 (0.53–2.11)	0.89 (0.66–1.20)
40–44	0.72 (0.53–0.97)*	0.57 (0.31–1.08)	1.02 (0.74–1.38)
45–49	0.54 (0.38–0.75)**	1.00 (0.46–2.18)	0.72 (0.52–1.00)
50–54	NA	0.68 (0.30–1.50)	NA
55–59	NA	0.76 (0.33–1.76)	NA
**Working Status**			
Not working	Reference	Reference	Reference
Working	1.10 (0.92–1.31)	0.92 (0.63–1.36)	1.80 (1.51–2.15)**
**Interaction Term**			
Rural*working	1.78 (1.15–2.75)*	0.94(0.41–2.18)	1.27(0.83–1.93)

** p<.001;

* p<.05.

§ This analysis was done among those women who know (heard of) the practice of FGM; we excluded women who did not know of FGM, did not belong to one of the 4 major ethnic groups, and the missing variables.

¥ This analysis was done among those women who either approve or disapprove the continuation of FGM; we excluded women who did not know of FGM, did not belong to one of the 4 major ethnic groups, and the missing variables.

The predictors of FGM approval (noted above) were also important in the model assessing relationships between socio-demographic variables and the outcome of FGM experience. In addition, working status and marital status were significant in this model. Married women were almost one and half [O.R. 1.31 (95% CI 1.08–1.58)] times more likely than women not married to have experienced FGM. Working women on the other hand were 1.80 (95% CI: 1.51–2.15) times more likely than non-working women to have experienced FGM. Interestingly, older women (ages 45–49 years old) were less likely to report experiencing FGM compared to women 15–19 years old.

Results from the 3 logistic regressions with the outcomes of attitude and practice of *gavage* are presented in Table 5. As with FGM, ethnicity, location, education, wealth, and age were significantly associated with approval of the continuation of *gavage*. In this analysis, Arab women and men were 4.96 (95% CI: 3.07–8.00) and 2.52 (95% CI: 1.53–4.18) times more likely to approve of the continuation of *gavage*, respectively, as compared to other ethnicities. Males from Hogh cha were 14.38 (95% CI: 6.60–31.31) times more likely to approve of the continuation of *gavage* as compared to males residing in Nouakchott. Also noteworthy is that any education and high wealth reduces the odds of approving the continued practice of *gavage*, as compared to no education and low wealth. Older women were less likely [O.R. 0.51 (95% CI: 0.38–0.68)] to approve of the continuation as compared to women between the ages of 15 and 19 years old. One interaction term was determined to be significant, whereby having a job and being located in a rural area almost doubled the odds [O.R. 1.67 (95% CI: 1.22–2.28)] of approving the continued practice of *gavage*.

**Table 5:** Multivariate analysis of factors related to the practice and attitude toward *gavage*.

	Favorable attitude for continuing *gavage*		Experienced *gavage*
	Women (n = 6,190)	Men (n = 1,803)	Women (n = 7,208)
	*Odds Ratio (95% CI)*	*Odds Ratio (95% CI)*	*Odds Ratio (95% CI)*
**Ethnicity**			
Other ethnic groups	Reference	Reference	Reference
Arab	4.96 (3.07–8.00) **	2.52 (1.53–4.18)**	17.46 (10.75–28.35)**
**Place of residence**			
Urban	Reference	Reference	Reference
Rural	1.47 (1.13–1.92) *	1.33 (0.69–2.56)	1.45 (1.12–1.89)**
**Wealth**			
Lowest	Reference	Reference	Reference
Second	0.76 (0.63–0.90)*	0.75 (0.54–1.04)	0.91 (0.77–1.08)
Middle	1.00 (0.70–1.43)	1.01 (0.60–1.71)	0.94 (0.68–1.28)
Fourth	0.63 (0.50–0.79)**	0.52 (0.32–0.84)*	1.19 (0.95–1.50)
Highest	0.58 (0.45–0.74)**	0.57 (0.34–0.95)*	1.23 (0.96–1.57)
**Education**			
No	Reference	Reference	Reference
Koranic	0.77 (0.64–0.93)*	0.81 (0.56–1.18)	2.13 (1.76–2.59) **
Prim. Education	0.64 (0.52–77)**	0.45 (0.31–0.66)**	1.27 (1.00–1.60)
Second. Education	0.35 (0.28–0.44)**	0.32 (0.22–0.47)**	1.05 (0.79–1.39)
High (University)	0.27 (0.15–0.50)**	0.13 (0.06–0.26)**	0.75 (0.41–1.35)
**Age**			
15–19	Reference	Reference	Reference
20–24	0.86 (0.70–1.06)	0.60 (0.40–0.92)*	1.43 (1.14–1.79)*
25–29	0.82 (0.66–1.02)	0.69 (0.46–1.05)	1.63 (1.28–2.07)**
30–34	0.75 (0.60–0.93)*	1.90 (0.52–1.56)	2.18 (1.66–2.87)**
35–39	0.51 (0.40–0.67)**	0.54 (0.30–0.95)*	2.73 (2.10–3.55)**
40–44	0.49 (0.37–0.64)**	0.46 (0.25–0.86)*	4.10 (3.03–5.55)**
45–49	0.51 (0.38–0.68)**	0.50 (0.26–0.98)*	4.14 (3.02–5.67)**
50–54	NA	0.60 (0.29–1.24)	NA
55–59	NA	0.55 (0.25–1.21)	NA
**Working Status**			
Not working	Reference	Reference	Reference
Working	0.84 (0.69–1.03)	1.56 (1.06–2.29)*	1.01 (0.85–1.20)
**Interaction Term**			
Rural*working	1.67 (1.22–2.28)*	0.75 (0.40–1.39)	0.69 (0.51–0.93)*

** p<.001;

* p<.05.

§ This analysis was done among those women who know (heard of) the practice of *gavage*; we excluded women who did not know of *gavage*, did not belong to one of the 4 major ethnic groups, and the missing variables.

¥ This analysis was done among those women who either approve or disapprove the continuation of *gavage*; we excluded women who did not know of *gavage*, did not belong to one of the 4 major ethnic groups, and the missing variables.

Results from the regression with the outcome of having experienced *gavage* were similar to those related to attitude towards *gavage*. Importantly, Arab women were over 17 (O.R. 17.46 (95% CI: 10.75–28.35)] times more likely to report having experienced *gavage*, as compared to other ethnicities. While education reduced the odds of approving the continuation of *gavage*, in this analysis having Koranic education doubled the odds [O.R. 2.12 (95% CI: 1.76–2.59)] of having experienced *gavage,* as compared to having no education. In this analysis, the interaction term between having a job and being located in a rural area decreased the odds [O.R. 0.69 (95% CI: 0.51–0.93)] of having experienced *gavage*.

### Overlap between FGM and Gavage

An investigation into the potential overlap between practices among women suggests that in general little overlap exists. One in five (19%) reported experiencing both *gavage* and FGM; 19% reported experiencing neither. Only 4% reported being force-fed, but not cut; 58% reported experiencing FGM, but not *gavage*.

## Discussion

Results from this study suggest that both FGM and *gavage* are actively practiced and approved of in Mauritania; these practices are unlikely to be discontinued in the near future without considerable effort to change cultural norms. Region-specific interventions should be developed to increase awareness about the public health and psychological dangers these practices pose. Given that Mauritania has already committed to protect the rights and welfare of children [30], this existing framework should be used to challenge these cultural norms. While these practices are illegal if they cause pain or suffering to children, perpetrators are not held accountable, likely the result of engrained tradition that encourages these practices [7], [31], [32]. It is also possible that by prosecuting offenders the practice would be perceived as a cultural attack and largely driven underground, rendering surveillance and the development of intervention to change cultural norms difficult.

The results of this study are important, as they constitute the first study around the practice and attitude of women and men toward FGM and *gavage* in Mauritania. These results might further serve as a base for developing evidence-based strategies to combat these harmful practices. Observations related to important predictors of FGM and *gavage* are discussed below, in the context of how best to protect the rights and welfare of children.

Both practices appear to be largely tied to ethnicity, although the ethnicities are different for FGM and *gavage*. FGM for example was highest among the Soninke and lowest among the Wolof, consistent with other regional studies [33]. *Gavage* however was most prevalent among Arab women. These observations provide a starting point for identifying which groups could benefit from increased awareness and education related to the public health and psychological dangers these practices pose. Awareness raising campaigns focused on behavior change communication could then be integrated into media and traditional information sources within regions most likely to practice the respective traditions.

While other studies have produced mixed results on associations between education and FGM, [3], [33]–[38], this analysis showed education to have a protective effect on attitude and practice of both FGM and *gavage*. Although the practice of *gavage* was positively associated with Koranic education, this finding is likely explained by the large proportion of Arab women with Koranic education confounding our results. Educators in collaboration with civil authorities should continue their focus on destigmatizing the refusal of these practices. Other interventions could target school-aged children, thus creating a generation with the information to question the merit of these practices.

One perplexing finding from this analysis was the seemingly contradictory role of age in relation to attitudes and practice of *gavage*. Older women were more likely to have experienced *gavage* than their younger counterparts, which might be expected if the prevalence was even higher in the past. However, positive attitudes toward the continuation of the practice of *gavage* were higher among younger women than older respondents. One explanation may be that these older women have experienced the health consequences of *gavage*, such as diabetes or cardiovascular diseases, and have thus received information linking these conditions to obesity; younger women may not have experienced any health consequences associated with obesity, or may not be targeted for chronic disease and healthy lifestyle information dissemination. A second explanation may simply be that women who have experienced gavage recognize that this is torture, and thus less likely to have a positive attitude regardless of the long-term health consequences. It is important that all age groups be targeted for strategic intervention, although the delivery mechanism will likely need to be tailored between age groups to improve effectiveness.

A second perplexing finding relates to the discordance between those that reported no disadvantages to the practice of FGM and those that favored its continuation. In this analysis, it was clear that some respondents (both male and female) reported disadvantages related to the practice of FGM yet favored its continuation. While the explanation is not clear from our data, it could be that Mauritania is a society with engrained and culturally sanctioned norms and values that directly influencs decision-making.

As these data were collected over a decade ago, information from other sources also provides useful insights into the problem of *gavage* in contemporary Mauritania. Indeed, there appears to be a movement toward an even more dangerous form of “fattening” young women to make them more desirable: the use of pharmaceutical drugs. A U.N. Population Fund official in Mauritania reported that: “for the little girls that are being force-fed, the practice is getting more dangerous; before they used camel milk, nowadays the girls are force-fed with chemicals used to fatten animals” [25], [39]. Similarly, a 2011 State Department report begins with encouraging news of a decrease in *gavage,* but ends with the same troubling mention of chemical substances being used to achieve a fattened state [40].

Despite the legislation banning harm to children, both FGM and *gavage* persist. Importantly, no one has ever been prosecuted for FGM or *gavage* in Mauritania. A prominent child rights lawyer in Mauritania is quoted as stating: “I have never managed to bring a case in defense of a force-fed child, as the politicians are scared of questioning their own traditions” [7]. A comprehensive strategy to combat FGM and *gavage* should adopt a community-led developmental-based approach to include a plan to empower women economically and politically, especially in rural areas, and a plan to reduce illiteracy. Such a strategy cannot be implemented without substantial involvement and participation of religious leaders, community leaders, women and both male and female youth. Religious leaders could convey the message that FGM and *gavage* are not mandated by religion; public health and medical professionals could explain the sexual psychological and reproductive health complications associated with the practices; women and youth associations could focus on discussing and disseminating correct information related to these practices; and the government must focus on protecting the rights and welfare of children through the enforcement of law. National policies and legislations may not change cultural behavior, but such mechanisms could create a framework for openly discussing the public health and human rights concerns of such practices.

This study has a few limitations worth mentioning. First, the analyses performed were done using cross-sectional data, thus precluding any causal inference. Second, the data were collected in 2000; although unlikely, given information from other sources, it is possible that the current situation related to FGM and *gavage* is different from 10 years ago. However, the recent DHS conducted in 2010 did not include *gavage*, making the data used in this study the only nationally representative survey data available that contains both practices. Lastly, given the sensitive nature of both FGM and *gavage* in the cultural and legal sense, these data may be prone to recall bias and bias related to self-reported activity.

In conclusion, this study lends insight into two culturally sanctioned practices that have implications for the protection of children. These results should serve as a basis for the development of targeted interventions and strategies to change cultural perceptions. Until *gavage* is explicitly stated to be illegal, and current FGM laws criminalizing the act are enforced, reducing these practices will be a difficult task and countless children will continue to be subjected to these cruel and harmful practices.
